# Story-linked item design in tablet-based assessment for preschool children: Insights from testing

**DOI:** 10.4102/ajopa.v6i0.154

**Published:** 2024-05-28

**Authors:** Rivca Marais, Louise Stroud, Cheryl Foxcroft, Johan Cronje, Jennifer Jansen

**Affiliations:** 1 Department of Psychology, Faculty of Social Science and Humanities, University of Fort Hare, Alice, South Africa; 2 Department of Psychology, Faculty of Health Sciences, Nelson Mandela University, Gqeberha, South Africa; 3 Higher Education Access and Development Services (HEADS), Nelson Mandela University, Gqeberha, South Africa

**Keywords:** tablet-based assessment, story-linked, gamification, developmental assessment, digital test development

## Abstract

This article provides a rationale for exploring the use of tablet-based assessment of children between the ages of 3 years and 5 years. The purpose of the study was to gain insights from young children’s digital test-taking performances and experiences to inform the digitalisation of developmental tests. A mixed method design was followed to collect both qualitative and quantitative data. Animated tablet-based items following a storyline were field-tested on a sample of 60 South African children. Results support the viability of a story-linked, tablet-based gamification approach for assessing children 5 years and under, and emphasise the need for documented strategies and item examples to guide innovative developmental assessment. Digital items showed a degree of responsiveness to various factors, suggesting a potential influence on test taking performance which contributes to the necessity of re-imagining item and test development in the digital age.

**Contribution:**

This study departed from the conventional path of following the predictable and conservative approach of test development taken so far of merely adapting existing measures to a digital format. By empirically assessing the efficacy of newly developed items designed specifically for a digital format, this article addressed the intersection of technology and psychological assessment of the preschool child in a South African context.

## Introduction

Technology is enhancing psychological test design and assessment by offering a range of possibilities through the incorporation of tablets and smartphones in testing which goes beyond converting paper-based testing materials to a digital format (McHenry et al., 2023). Standardised assessments are changing as digital platforms expand, pushing the boundary between a virtual and real world with a touch screen interface (Hubber et al., 2016; McEvoy & Woitaszewski, 2014). To date, there has been a notable acceleration in the use of computerised testing, primarily driven by the widespread integration of technology in the field of psychology during the coronavirus (COVID-19) pandemic (Martínez-Cengotitabengoa et al., 2022). This broader adoption of technology in the psychological sphere has further emphasised the need for digital assessment to align with the evolving demands of best practice. Digital test adaptation and development have predominantly focussed on measures for adults suggesting that adapting digital items is generally easier for individuals aged five years and above (Drozdick et al., 2016; Marais et al., 2020; Pade, 2014).

Pearson’s Clinical Assessment Group was among the first to embark on adapting ability-based measures for children, such as the Wechsler Intelligence Scale for Children–Fifth Edition (WISC-V), the Wechsler Pre-school and Primary Scale of Intelligence™–Fourth Edition (WPPSI-IV) for tablet use (Daniel 2013a; Pade, 2014). This practice progressed to multi-domain child developmental measures including the Ages and Stages Questionnaire–Second Edition (ASQ-2), The Raven’s Progressive Matrices (R-PDQ), The Vineland Adaptive Behavior Scales (Vineland), The Bayley Scales of Infant and Toddler Development–Third Edition (Bayley-3), and NEPSY–Second Edition (NEPSY-II) (Komanchuk et al., 2023; Pade, 2014). From more than 33 multi-domain child developmental measures, currently only these five measures have been adapted digitally (Komanchuk et al., 2023). However, these tests are digital adaptations that mostly replicate the traditional paper-based method of testing as the tablet mainly functions as replicas of paper-based stimuli (Björngrim et al., 2019). These tests are not exclusively designed for the digital mode and lack innovative features like animation and game-based elements, aiming to preserve construct equivalence with the traditional test version (Drozdick et al., 2016; Krkovic et al., 2014; Pade, 2014). Further progression in this field focussing on child developmental measures tailored exclusively for digital use still lacks substantial interactivity despite being developed specifically for digital formats (Pitchford & Outhwaite, 2016). These existing trends highlight the challenges related to the costly undertaking of test development and the incorporation of innovative aspects including the scarcity of supportive digital item examples and limited guidelines in the field, all of which tend to stifle innovative test development (Marais, 2020; McHenry et al., 2023). The above-mentioned challenges are particularly evident in lower-income countries, where, despite the global progression of technology in assessments, there is a discernible lag in research focus, development, and the incorporation of innovation in assessment practices (Fernald et al., 2017). According to Foxcroft and Roodt (2018), the prevailing trends lean towards test adaptation rather than test development in lower-income countries. Furthermore, the slow pace of development and innovation in assessment tools in lower-income countries is evident in the limited availability of adaptive and interactive digital assessments.

Presently, there is no comprehensive and diagnostic computer- or tablet-based cognitive measure known to have been developed or adapted for young children in South Africa. The only developmental measure developed in South Africa, featuring digital components, is the Early Learning Outcomes Measure (ELOM) (Snelling et al., 2019). The administration follows traditional testing procedures but offers a digital scoring system and a digitally accessible manual for users (Raikes et al., 2019). There is thus a critical gap in knowledge pertaining to the assessment of children under the age of 5 years using innovative technological aspects especially in lower- and middle-income countries (Drozdick et al., 2016).

To bridge this gap, the study sought to investigate the insights gleaned from the digital test-taking experiences and performance of a sample of 60 children between the age range of 3 years to 5 years on an experimental digital item set that follows a storyline and comprises cognitive, animated game-based items. This article reports on this process, which is the third testing phase within a larger project, that consisted of four phases aimed at developing guidelines for the development of cognitive assessment items that are designed exclusively for a digital platform (Marais et al., 2020). The results offered valuable insights and challenges into the performance of participants from different age groups, as well as identifying patterns in the performance of the different age cohorts. Computerised tracking of performance, together with data obtained from observations, as well as video recordings further enriched the understanding of participants’ interactions with the digital tasks.

## Methods

### Research design

A convergent parallel mixed method design was employed to gather comprehensive insights into the analysis and experience of performance on story-linked cognitive test items for assessing children in the third phase as reported in this article (Creswell, 2014). Both qualitative and quantitative data were collected concurrently, analysed separately, and then compared during the interpretation of the data. Quantitative data were gathered through testing of the digital items as well as computerised tracking of performance which provided numerical insights into performance metrics. Concurrently, qualitative data were collected through test observations and video recordings to enrich the understanding of children’s experiences and behaviours during the testing process.

### Sample

The study used non-probability, purposive sampling. This type of sampling allowed the researcher to select the sample group on the basis of specific characteristics which included age (3 years 0 months to 5 years 11 months), location (Nelson Mandela Bay Municipality and Raymond Mhlaba Municipality District), normality (deemed to have had a typical, uncomplicated birth and an on-course, uneventful developmental history with milestones reached on time), language (language of tuition, English), attending a fee-paying preschool or a daycare centre, and exposure to different forms of technology (have played on a tablet or touch screen smartphone before). A sample of 60 children between the ages of 3 years and 5 years residing in South Africa were selected.

Children from three age groups were sampled for the study. The number of participants aged 3 years and 4 years were the same with 21 (35%) each and the sample size for participants aged 5 years was 18 (30%). The mean age was M = 3.95 years, and the standard deviation (s.d.) = 0.81 years. The mean age suggests that, on average, participants were close to 4 years old while the small s.d. indicates low age variability. The sample consisted of 34 (57%) girls and 26 (43%) boys. All 60 children had an uneventful medical history and met their developmental milestones within expected time frames. Access to a computer was reported by 50% of the sample, while 75% had access to a smartphone, and 70% had access to a tablet. The geographical distribution of the sample included 36 children (60%) from five different playschools and preschools within the Nelson Mandela Bay Municipality district, and 24 children (40%) from three different playschools and preschools situated in the Raymond Mhlaba Municipality district. Of the participants included in the study, 21 (35%) attended a playschool, while 39 participants (65%) attended a preschool. All 60 (100%) participants of the sample received tuition in English. According to the biographical questionnaire, 90% of the sample demonstrated ease in operating touch screen devices. Overall, it was concluded that the participants were comfortable with technology, especially touch screen devices.

### Data collection instruments

#### Biographical questionnaire

A biographical questionnaire was designed for the study to gather information on participants’ age, location, language, developmental history, preschool or playschool attendance as well as details regarding their technological exposure and usage. The completion of the biographical questionnaire was undertaken by the parents.

#### Cognitive digital item set

To collect the relevant data, a newly developed application consisting of seven digital items that followed a storyline with animation and gamification elements was utilised to test the participant’s digital test-taking performances and experiences. Each digital item included animation to convey the item instructions. Animation was also used to assist with item connectivity as well as to emphasise certain aspects of the item. The focus of this article reports on three of the seven items to enable a more in-depth discussion. The selection of item reporting involved presenting the items with varying levels of interactivity: one with a high level of activity, specifically, Shoes on Shelf; a medium-level interactive item, namely, Price Tag; and the item with the lowest interactivity level in the set, Most Shoes. These were chosen to address identified gaps in knowledge as research evidence suggests psychometric measurement challenges particularly with interactivity levels in children younger than five years (Drozdick et al., 2016). The items were contained in an application (app) that required installation on a tablet. The study used a Samsung device with a 10-inch screen as suggested by usability studies that recommend larger screens for educational purposes (Daniel, 2012a, 2012b, 2012c, 2012d, 2012e, 2012f; 2013a, 2013b; Daniel et al., 2014). Each digital item is designed to generate a numerical output through dichotomous scoring indicating pass or fail for a specific developmental task. A narrative thread connected the seven items and involved a donkey and a chicken embarking on a shopping adventure with which most South African children could identify. The digital items included music, sound effects, animation, and talking characters together with other interactive elements, such as a star button to indicate completion of an item. The child was required to assist two characters to complete tasks by dragging or dropping objects or tapping on items to indicate their choices. The characters were designed to interact with the children by greeting them and providing instructions for completing the item. Further programming allowed for the collection of data on human-computer interaction, which involved quantitative tracking of each child’s attempts. This tracking included aspects like measuring the time taken to complete the activity and analysing repetitive touch responses.

**FIGURE 1:**
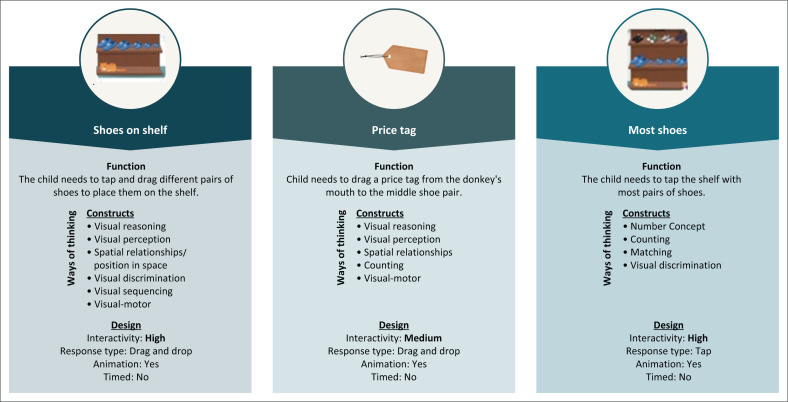
Overview of functions, underpinning constructs and design concepts.

The three items focussed on in this article were underpinned by the cognitive construct domain of ways of thinking. Ways of thinking involve skills essential for comprehending and organising information, such as problem solving, reasoning, visual perception, and numeracy (Stroud et al., 2016). This domain is among the most frequently used construct domains utilised by child developmental measures (Marais, 2020). As children utilised response types like drag-and-drop, as well as tap responses to interact with the touch screen, a visual-motor component was needed for each of the items. Figure 1 illustrates the functions, underpinning constructs, and design concepts for each of the reported digital items.

### Procedure

Every child had the opportunity to familiarise themselves with tablet touch responses before the administration of the digital items began. Registered psychologists and psychologists-in-training received training in the administration of digital items. Participants were assessed at three different centres, and apart from travel expenses there were no additional costs involved. On completion of the testing, parents received a brief qualitative report on the child’s performance. Two free applications with high star ratings and unrelated to the study’s storyline were downloaded from the Google Play Store and acted as practice examples of the touch responses required. One app utilised a tap response to pop balloons, while the other required a drag-and-drop touch response to manipulate puzzle pieces. Both practice applications were provided to every child and additional practice attempts were offered when necessary. Once the participants were able to execute the tap response as well as the drag-and-drop response in terms of accuracy, speed and precision, the digital assessment items were administered. Additional data were collected during the assessment using the tablet. This included the capture of quantitative performance metrics, such as the time taken to complete each item and the number of repeated touch responses. This supplementary data provided qualitative insights into the child’s performance and user experience without influencing the pass or fail scoring of the child. Furthermore, all testing sessions were video recorded. The video-recordings together with the computerised tracking of performance provided an extra benefit by providing information on how participants interacted with the digital items. This process aligns with several studies that found video recordings to be invaluable in all their digital assessment studies (Daniel, 2012a; Daniel et al., 2014; Drozdick et al., 2016; Raiford et al., 2016; Markle et al., 2011).

### Data analysis

The quantitative data of phase three first involved descriptive statistics. This was done by computing the frequency and percentage distribution of different age groups (3, 4, and 5 years) on passing or failing for each digital item. In addition, item difficulty (*p* = 0.05) was calculated for each item, for both the total sample and within each age group by dividing the number of participants who answered each item correctly by the total number of participants who responded. Thereafter, a Chi-square test was used to determine whether a significant difference was found in the pass or fail performances across age groups 3, 4, and 5 years on the three items. Because of the categorical nature of the data and the absence of mean scores, further post hoc analysis involved calculating standardised residuals to discern specific cell deviations within the contingency table (Sharpe, 2019). Further item analysis was conducted by using the data obtained through computerised tracking. Mean scores from this data were analysed for the time taken to complete each item and repetitive touch responses using a one-way analysis of variance (ANOVA). This computation was conducted to ascertain whether statistically significant differences exist between the means of the age groups regarding the time taken to complete tasks and repetitive touch responses. Where significant differences in group means were found for these two human-computer interaction variables, an additional post hoc comparison was performed using the Bonferroni post hoc test. In addition to the quantitative analysis, qualitative data from clinical observations and video recordings were thematically and visually analysed. This added further richness to the quantitative analysis. Qualitative data obtained from test administrators and video recordings were thematically analysed by following the six steps advocated by Braun and Clarke (2021).

### Ethical considerations

Ethical approval (ethics number: H16-HEA-PSY-042) was granted by the Nelson Mandela University’s Research Ethics Committee (Human). Informed consent was diligently acquired from parents and caregivers, ensuring data confidentiality and inclusive, discrimination-free selection criteria, prioritising participant privacy through rigorous data protection measures. As children were deemed to be below the age threshold for providing assent, the research protocol involved obtaining informed consent from parents for both testing and video recording of their child.

## Results

### Quantitative results

#### Item analysis: Shoes on Shelf

The quantitative findings revealed distinct performance variations among the 3-, 4-, and 5-year-old age groups in relation to the Shoes on Shelf item. This item is ranked as the most difficult of the three items as only 33% of the total participants passed. As can be seen from Table 1, the 5-year-old age group performed better than the 4- and 3-year-old age groups on this item. The 4-year-old performed better than the 3-year-old but worse than the 5-year-old. This item was particularly difficult for the 5-year-old age group to pass, as 90% of the 5-year-olds failed this item. The Chi-square analysis (χ^2^ = 8.46, *df* = 2, *p* < 0.01) indicated significant performance differences between age groups on this item. The subsequent post hoc analysis indicated that the item was too difficult for the 3-year-old age group, with a standardised residual of –1.9, signifying a deviation below the expected frequency. A standardised residual of 0.8 was obtained for the 4-year-old age group which indicates a deviation above the expected frequency. Similarly, the 5-year-old age group obtained a standardised residual of 1.2 which also suggests a deviation above the expected frequency. The impact of the 3-year-old age group on the overall Chi-square result was particularly notable for this item, indicating a significant deviation in their performance on the Shoes on Shelf item from what would be expected by chance.

**TABLE 1:** Summary frequency for shoes on shelf.

Frequency response	Age (years)	Pass	Fail	Total
Count	3	2	19	21
Row percent	-	10	90	100
Count	4	9	12	21
Row percent	-	43	57	-
Count	5	9	9	18
Row percent	-	50	50	100
Count	All groups	20	40	60
Row percent	All groups	33	67	100

Note: Ways of thinking: visual reasoning, visual perception, spatial relationships/position in space, visual discrimination, visual-motor. *Interactivity: high; Response type: drag-and-drop; Animation: yes; Timed for scoring: no. df* = 2; Chi-square = 8.46; *p* = 0.01.

#### Item analysis: Price tag item

Results seen in Table 2 indicate that 72% of the total sample passed this item with 95% of the 4-year-olds passing this item in comparison with 5-year-olds where the pass rate was 72% on the Price Tag item and 3-year-olds who had a pass rate of 48%.

**TABLE 2:** Summary frequency for price tag.

Frequency response	Age (years)	Pass	Fail	Total
Count	3	10	11	21
Row percent	-	48	52	100
Count	4	20	1	21
Row percent	-	95	5	100
Count	5	13	5	18
Row percent	-	72	28	100
Count	All groups	43	17	60
Row percent	All groups	72	28	100

*Source*: Marais, R., Stroud, L., Foxcroft, C., & Cronje, J. (2020). Guidelines for story-linked digital item design for the cognitive assessment of pre-school children. Unpublished doctoral dissertation. Nelson Mandela University, South Africa

Note: Ways of thinking: visual perception, spatial relationship, numeracy, counting, visual-motor. *Interactivity: medium; Response type: drag-and-drop; Animation: yes; Timed for scoring: no. df* = 2, Chi-square = 11.73, *p* = 0.00.

The Chi-square analysis (χ^2^ = 11.73, *df* = 2, *p* < 0.00) indicates significant performance differences between age groups on this item. The subsequent post hoc analysis indicated that the 3-year-old age group again faced considerable difficulty, as evidenced by a standardised residual of −1.3, indicating a deviation below the expected frequency for successfully completing the item. In contrast, 4-year-olds demonstrated a proficiency level beyond the expected frequency reflected in a standardised residual of 1.3. Similarly, 5-year-olds obtained a standardised residual of 0, suggesting performance in line with the anticipated frequency on this item.

#### Item analysis: Most Shoes

Most Shoes was recorded as the item that was the easiest to complete with only 22% of the sample failing this item. The interactivity and animation of this item was low and only required a tap response. Results presented in Table 3 indicate that 89% of five-year-olds passed the item while the pass rate for the four-year-old age group was 81%. A further decline in the pass percentage to 76% was recorded for the three-year-old age group.

**TABLE 3:** Summary frequency for most shoes.

Frequency response	Age (years)	Pass	Fail	Totals
Count	3	14	7	21
Row percent	-	67	33	100
Count	4	17	4	21
Row percent	-	81	19	100
Count	5	16	2	18
Row percent	-	89	11	100
Count	All groups	47	13	60
Row percent	All groups	78	22	100

*Source:* Marais, R., Stroud, L., Foxcroft, C., & Cronje, J. (2020). Guidelines for story-linked digital item design for the cognitive assessment of pre-school children. Unpublished doctoral dissertation. Nelson Mandela University, South Africa

Note: Ways of thinking: visual discrimination, visual matching, number concepts, counting. *Interactivity: low; Response type: tap; Animation: yes; Timed for scoring: no. df* = 2, Chi-square = 2.95, *p* = 0.23.

The Chi-square analysis (χ^2^ = 2.95, *df* = 2, *p* < 0.23) indicated that the Most Shoes item presented an appropriate level of challenge for 3-year-olds but was relatively easy for 4 and 5-year-olds. However, the difference in performance between the 5-, 4- and 3-year-old age groups was not statistically significant.

#### Computerised tracking of performance

The Shoes on Shelf item exhibited a mean completion time of 54.50 seconds (s.d. = 31.32 seconds) for the total sample as can be seen in Table 4. The one-way ANOVA indicated a significant difference for the completion time among age groups with F(2, 53) = 4.02, *p* = 0.02. Comparisons were made among the three age groups using the Bonferroni post hoc test. The results indicated that the mean time taken by 3-year olds to complete Shoes on Shelf (M = 67.37, s.d. = 33.91) was significantly different (*p* = 0.02) to the mean time taken by 5-year olds on the item (M = 39.11, s.d. = 23.94). However, the average time taken by the 4-year old age group to complete Shoes on Shelf (M = 53.95, s.d. = 29.39) did not differ significantly from the average time taken by the 3-year old age group (*p* = 0.48). It is thus evident that the 5-year-old age group performed the strongest and completed this item in the fastest time while the 3-year old age group performed the weakest and recorded the slowest time. The 4-year-old age group was faster than the 3-year-old age group but slower than the 5-year-olds but the difference was not significant (*p* = 0.43).

**TABLE 4:** Analysis of variance: Computerised tracking of performance.

Interface response	Year group	$\bar{\text{x}}$	s.d.	*df*	F	*p*
**Shoes on shelf**						
Time to complete *(in seconds)*	3	67.37	33.91	2	4.02	0.02
	4	53.95	29.39	-	-	-
	5	39.11	23.94	-	-	-
Total	-	54.50	31.32	-	-	-
Repeated touch	3	12.55	12.42	2	4.18	0.02
	4	7.65	9.74	-	-	-
	5	3.31	2.92	-	-	-
Total	-	8.16	10.11	-	-	-
**Price tag**						
Time to complete *(in seconds)*	3	31.93	15.51	2	3.42	0.04
	4	27.12	14.96	-	-	-
	5	19.72	9.93	-	-	-
Total	-	26.72	14.54	-	-	-
Repeated touch	3	5.25	5.79	2	4.18	0.02
	4	3.45	4.74	-	-	-
	5	0.88	1.09	-	-	-
Total	-	3.36	4.77	-	-	-
**Most shoes**						
Time to complete *(in seconds)*	3	14.24	5.85	2	0.20	0.82
	4	15.18	13.42	-	-	-
	5	13	9.65	-	-	-
Total		14.22	10.01	-	-	-
Repeated touch	3	3.2	3.81	2	0.92	0.40
	4	2.65	3.82	-	-	-
	5	1.63	2.45	-	-	-
Total	-	2.55	3.47	-	-	-

s.d., standard deviation; *df*, degrees of freedom.

Computerised tracking of performance revealed that the total sample repeatedly touched the screen eight times on average with the administration of Shoes on Shelf (M = 8.16, s.d. = 10.11) The one-way ANOVA indicated a significant difference for the time to complete among age groups with F(2, 53) = 4.18, *p* = 0.02. A subsequent post hoc test confirmed a significant difference (*p* = 0.02) in the mean repeated touch responses between the 3-year old and 5-year old age group. On average, 3-year-old participants touched the screen significantly more (M = 12.55, s.d. = 12.42) compared to 5-year-olds (M = 3.31, s.d. = 2.92). No significant difference (*p* = 0.34) was observed in the mean repeated touch responses between the 3-year-old age group and 4-year-old age group. Similarly, there was no significant difference in the mean touch responses between the 4-year-old and 5-year-old age groups (*p* = 0.55).

Significant results were further obtained for both the time taken to complete the item and the frequency of repeated touch responses for the Price Tag item. In Table 4, data presented indicates that the item took on average 26.72 s to complete (s.d. = 14.54). The one-way ANOVA indicated a significant difference for the time taken to complete this item among the age groups with F(2, 53) = 3.42, *p* = 0.04. A further post hoc test indicated that the mean score obtained by the 3-year-old children for the time taken to complete the Price Tag item (M = 31.93, s.d. = 15.51) was significantly different (*p* = 0.04) to the mean score obtained by the 5-year-old children on this item (M = 19.72, s.d. = 9.93). No significant difference was obtained between the 3- and 4-year-old age groups (*p* = 0.84), as well as between the 4-year-old and 5-year-old (*p* = 0.36) age groups. Overall, the time taken to complete the Price Tag item suggests a significant decrease in the time taken as the child’s age increased concurring with the finding for Shoes on Shelf.

The total sample repeatedly touched the screen three times on average with the administration of Price Tag (M = 3.36, s.d. = 4.77). The one-way ANOVA indicated a significant difference for repeated touch responses among age groups assessed on this item F(2, 53) = 4.18, *p* = 0.02. The average time to complete the item by 3-year-olds was 5.25 s (s.d. = 5.79). Among the 4-year-old age group, the average time decreased to 3.45 s (s.d. = 4.74). The 5-year-old age group exhibited the shortest completion time, with an average of 0.88 s (s.d. = 1.09). A further post hoc test indicated that on average the 3- year-old age group touched the screen significantly more when compared to the older 5-year-old participants (*p* = 0.02). No significant differences were found between 3- and 4-year-olds (*p* = 0.64 and between 4-and 5-year olds (*p* = 0.29).

The completion time for the third item reported on namely, Most Shoes, exhibited a mean duration of 14.22 s and a s.d. of 10.01 for the total sample, as evidenced by the data presented in Table 4. The one-way ANOVA results revealed that there were no statistically significant differences in the time taken to complete the specified item across different age groups F(2, 53) = 0.20, *p* = 0.82. Furthermore, children repeatedly touched the screen two and a half times (M = 2.55, s.d. = 3.47) during the completion of this item. Again, no significant difference was found between age groups for repeated touch on this item as indicated by the one-way ANOVA with F(2, 53) = 0.92, *p* = 0.40.

### Qualitative results

Clinical observations recorded by test administrators together with video recordings enabled the researcher and reference group to observe as to how administrators and test-takers interacted with the three digital items presented in this paper. This information could be used to assist in refining items and guiding improvements in interface design. The thematic analysis of the video observations and reported clinical observations of the three items yielded the following qualitative findings.

#### Theme 1: Storyline

Qualitative observations indicate that all three age groups engaged with the storyline from the outset of the first item, namely Shoes on Shelf. It was noted that the younger children engaged more with the storyline through fantasy play, intimately interacting with the characters, while older children responded in a more realistic manner, following the instructions of the characters.

#### Theme 2: Item design and format

Shoes on Shelf initiated extensive exploration of the item design by repeated screen tapping as evidenced in video recordings and clinical observations. Most children showed a preference for the digital format presented by the practice examples involving tapping (popping balloons) over the Shoes on Shelf item which demanded greater mental effort and concentration.

Further design-specific issues that were identified across the three items included the need for larger touch-sensitive areas around smaller objects. This recognition stemmed from the observation that limited responsiveness impeded lift activation during drag movements, requiring greater visual-motor precision to execute the activation of the small hotspot. This was noted for both the Shoes on Shelf item and the Price Tag item which required a drag-and-drop touch response. A further design aspect that was flagged was how the design of the item influenced the decision-making process of children with the completion of the Most Shoes item. Participants were required to select the shelf with the most shoes and were presented with a choice between blue shoes featured in previous scenes and a shelf displaying different shoes. The qualitative findings suggested that the participants tapped the shelf with the familiar blue shoes instead of strictly adhering to the given instruction of identifying which shelf had the most shoes.

#### Theme 3: Visual motor maturity

The Shoes on Shelf item demanding intricate ‘drag-and-drop’ interactions, required substantial visual-motor effort. Notably, participants with less developed fine motor control encountered challenges when executing the drag-and-drop movement. This was observed for both the Shoes on Shelf item and the Price Tag item. Specifically, the observed distance for the drag movement influenced performance. When the drag-and-drop movement exceeded the comfort level for many participants, it led to a focus on motor manipulation rather than the intended task. This was noted for both the Price Tag object and the Shoes on Shelf item. Both items required an extended drag-and-drop distance resulting in repeated attempts and reduced persistence, as the participants hesitated to move the price tag object again after it reached the shelf, irrespective of the correct placement. Furthermore, especially during video analysis of the Price Tag item observations revealed that the flat tablet placement contributed to the child’s hand obstructing the row of shoes during a continuous drag action over a long distance, which could have influenced the participants’ visual focus. No visual-motor influence was flagged for the Most Shoes item as this item did not require complex visual-motor dexterity.

## Discussion

Results of this study revealed fine-grained differences in the difficulty levels of digital test items among 3, 4, and 5-year-old children for the Shoes on Shelf item and the Price Tag items. This finding suggests an alignment with developmental theory on the role of biological maturation in child development (Case, 1998; Fischer & Bidell, 2006; Piaget, 1953). Both these items required a drag-and-drop touch response and were significantly difficult for the 3-year-old age group as the numerous drag-and-drop responses were found to be a challenging motor task for most children in this age group. Furthermore, significant differences between the 3- and 5-year-old age group, particularly in terms of the time taken to complete and the frequency of repeated touch responses for items requiring a drag-and-drop touch response were recorded. In Piaget’s preoperational stage, children typically exhibit concrete thinking, often fixating on a single aspect of an object (Watts et al., 2013). This cognitive pattern may have contributed to repeated touch responses, suggesting that children were focussed on the feedback of touch. Cognitive capacity also increases as cognitive development takes place and therefore the child’s ability to regulate their impulses improves as their cognitive capacity increases. Conversely, the absence of significant repeated touch responses between the 4-year-old group and the other two age groups, both for Shoes on Shelf and Price Tag, suggests that there are more distinct disparities in responses within a broader age gap, such as between 3- and 5-year-olds. Additionally, the lack of significant results between age groups for Most Shoes, once more, highlights the influence of biological maturation on performance across different age groups. This item entailed minimal interactivity and visual-motor input with only a tap response requiring less visual-motor maturity. Video recordings indicated that previous exposure to other items could have influenced this task as children may have tapped the shelf with familiar shoes rather than following the instructions. The lower visual-motor demand of this item resulted in fewer repeated touch responses, with no significant differences observed across all age groups, including the anticipated larger age gap between 3- and 5-year-olds. Furthermore, manipulating smaller items on the screen was also challenging for the 3-year-old group necessitating a larger hot spot around the objects to account for the children’s developing visual-motor skills. The inability to move objects or the difficulty in placing objects further influenced children’s persistence. Some gave up while others’ working memory appeared to be overloaded as the goal of the item was lost during the completion of the item (Case, 1992).

These results imply that the drag-and-drop function is better suited for the child older than four when utilised in psychological assessment measures and aligns with existing literature (e.g., Drozdick et al., 2016). If used with a child younger than four the results indicate that the design should consider avoiding multiple drag-and-drop requirements to complete the item. The distance required to drag the item should also be shorter. It was found that multiple drag-and-drop requirements across longer distances increased the visual-motor demand for the child, thereby interfering with the intention of the item, especially when the main construct measured was not visual-motor ability.

Notably, when comparing the findings among the 4-year-old to the 5-year-old age group on the Price Tag item, a positive maturation curve was not observed. Standardised residuals indicated that the 4-year-old age group performed better than expected on the Price Tag item. Despite this result, video recordings revealed greater visual-motor maturity by the 5-year-old age group as they showed better fine-motor control and precision. Further exploration highlighted that the Price Tag item required less visual-motor input compared to the Shoes on Shelf item. The 4-year-old child made more drag-and-drop movements, placing the Price Tag closer to the row of shoes, potentially facilitating an easier identification of the middle shoe. This contrasted with the 5-year-old group who made one continuous drag-and-drop movement over a longer distance. Additionally, the flat placement of the tablet on the table appeared to influence this item as the child’s hand obstructed the row of shoes during a continuous movement, affecting the older child’s ability to maintain a visual mark on the middle shoe pair. Piaget’s notion of perceptual centration in preoperational children may explain this, indicating a centration on mastering the drop and drag response. Results from the item analysis of Shoes on Shelf and the Price Tag item suggest that digital items are sensitive to digital effects, with young children being particularly vulnerable to these design issues as they are still maturing in their cognitive and fine motor skills. Such digital effects have the potential to influence the accuracy and reliability of the measurement of the test construct.

Qualitative data echoed the importance of child-centred usability and age-targeted item development which included test add-ons such as practice examples. Participants expressed a preference for practice examples characterised by lower mental effort and higher interactivity compared to the test items which were particularly noted for Shoes on Shelf. This preference might be associated with children’s prior digital experiences as suggested by information from the biographical questionnaire indicating that most children in the sample are accustomed to navigating the tablet at their own pace. However, the observed findings are more likely a result of differences in the practice examples, which did not measure the same constructs as the test and had distinct design and interactivity levels. Maintaining this consistency is essential for accurately assessing targeted cognitive abilities and minimising the potential influence of unrelated factors, such as experience with the digital world, on test performance (Foxcroft & Roodt, 2018). Qualitative results further revealed that children prefer uninterrupted play, high interactivity, and instant gratification when engaging with digital items. These preferences are important considerations for digital test development and validity, contributing to a more authentic assessment of cognitive abilities that mirrors real-world tasks. In addition, designing a test that effectively maintains a child’s engagement and interest contributes to a more accurate reflection of their developmental capabilities as they are more likely to engage actively with genuine intent. However, incorporating these preferences introduces additional considerations, such as cognitive and visual-motor maturity as well as item and format design, which add complexity to ensuring construct purity. Additionally, the study’s observations pointed out the potential usefulness of the digital assessment’s storyline, captivating children from the beginning and accommodating diverse age groups. It allowed younger children to engage in fantasy play while prompting older children to respond realistically to the characters’ instructions. The adaptable and engaging storyline also facilitated sustained attention and smooth transitions between items and provides further support for the utilisation of a story approach for tablet-based assessment for preschool children.

In summary, the present study acknowledges four main limitations. Firstly, the development process faced constraints in terms of budget, impacting the number and complexity of digital items created as well as to create practice examples that fit the constructs and design aspects of the digital items. Secondly, limitations imposed by fieldwork and sample constraints, including delays in tablet delivery and a non-probability sampling method, influenced the study’s sample size, generalisability, and ability to control certain variables. Thirdly, limitations in the digital set of items imply improvements to be made when designing digital items to enhance functionality and user experience. Fourthly, the absence of comparisons with performance on a pre-existing measure designed specifically for the digital format is a further limitation.

## Conclusion

This study represents one of the initial developmental assessment investigations aimed at collecting design-relevant information through the testing of digital items explicitly designed for tablets. This was achieved by developing animated, game-based items centred around a story or theme. The research results demonstrate substantial support for a story-linked, tablet-based gamification approach for assessing 21st-century children. The study further found that it is possible to design game-based digital items for ages three, four, and five. The incorporation of computerised tracking of performance enhanced clinical observations. This suggests promising clinical utility by potentially flagging significant variations in the time taken to complete the measure or excessive touch responses, thereby aiding in test result interpretation. However, it underscores the importance of age-targeted designs, touch responses, and interactivity levels in digital test design to accommodate the authentic engagement of the test-taker. It is also imperative to keep in mind and reduce potential design effects while accommodating the cognitive and developmental abilities of each age group in digital test development. Striking a balance between these factors becomes imperative, as it juxtaposes real-world, authentic assessments with the necessity for sound psychometric attributes. As there is an absence and lack of depth of digital item examples of specific constructs to use as a guide or clone, the importance of documenting the development of digital measures for tablet-based assessments of 3–5-year-old children is stressed. The results highlighted unanswered questions, that if answered could push the boundaries of psychometric assessment to meet the evolving needs of young children. Further research in the areas of exploring gamification, the impact of digital literacy on test performance, diverse item types and technologies, the influence of background music, and assessment for children with special needs is suggested. Another consideration worth noting, linked to the story approach is how one could assign a ‘mark’ to performance on the elements that jointly make up a story, and not just each item individually. It is anticipated that AI-based algorithms might have to be developed to evaluate performance on the story. Overall, far more research addressing questions regarding the essential psychometric properties of technology-based items is needed.
